# Supplementary material for: Interrater reliability and agreement of the NEUMOBACT checklist about infection-prevention performance of intensive care nurses in simulation-based scenarios
Source: PLoS One. 2024 Dec 31;19(12):e0313175. doi: 10.1371/journal.pone.0313175 (PMC11687784; doi:10.1371/journal.pone.0313175)
Supplement: S1 File — (DOCX) [file pone.0313175.s002.docx]

S1 File: Description of the simulation stations used in the OSCE-format SIMULAZERO course.

SIMULAZERO: simulation workshop for updating knowledge and skills in the prevention of ventilator-associated pneumonia (VAP) and catheter-related bacteraemia (CRB). Zero Projects.

| Table 1. OSCE summary | |
| --- | --- |
| Description | Circuit with three stations and short simulation scenarios to assess the technical and non-technical skills of ICU nurses in the VAP and CRB prevention. |
| Learning objectives | 1. To assess the technical skill of assisting the doctor performing the central venous catheter (CVC) insertion 2. To assess the technical skill of performing open- and closed-system endotracheal suctioning 3. To assess the non-technical skill of decision-making when caring for a patient with mechanical ventilation and CVC |
| Stations | Prebriefing: create a psychologically safe environment, provide instructions on materials available and their operation, roles to be played at the scenario, a fictional contract and confidentiality documentation (15 minutes)   - Station 1: central venous catheter insertion (10 minutes) - Station 2: endotracheal suctioning (10 minutes) - Station 3: patient care (10 minutes)   A maximum of 15 minutes should be allowed for change between stations  Debriefing: feedback on activities and decisions taken during the simulation at each station Self-assessment/evaluation with the NEUMOBACT instrument (30 minutes) |
| Duration | 90 minutes |
| Participants | Maximum number of participants at each station: 2  Maximum number of participants in the OSCE: 6 |

| Table 2: Description of the OSCE stations | |  |  |
| --- | --- | --- | --- |
|  | Learning objective | Participants’ roles | Actors |
| Station 1 (CVC): Central venous catheter insertion | To assess the technical skill of assisting the doctor performing the CVC insertion | Two ICU nurses | Confederated actor simulating an ICU junior doctor preparing to insert a CVC for the first time |
| Station 2 (ETS): Endotracheal suctioning | To assess the technical skill of performing open- and closed-system endotracheal suctioning | Two ICU nurses | Confederated actor simulating a nursing associate (NA) who assists the nurse in the ETS technique |
| Station 3 (PC): Care of patients with mechanical ventilation (MV) and CVC | To assess the non-technical skill of decision-making when caring for a patient with MV and CVC | Two ICU nurses | Confederated actor simulating an NA to enact the programmed events in the scenario. |

The clinical case details, actors’ scripts, equipment and consumables are described in the SIMULAZERO course [1].

[1] Raurell-Torredà M, Zaragoza-García I, Aliberch-Raurell AM, Sánchez-Chillón J, Torralba-Melero M, Arrogante O, et al. SIMULAZERO: taller de simulación para actualizar conocimientos y habilidades en la prevención de la neumonía asociada a ventilación mecánica y bacteriemia relacionada con catéter (Proyectos Zero). Enferm Intensiva. September 2022;33:S45-55
