# Supplementary material for: Interrater reliability and agreement of the NEUMOBACT checklist about infection-prevention performance of intensive care nurses in simulation-based scenarios
Source: PLoS One. 2024 Dec 31;19(12):e0313175. doi: 10.1371/journal.pone.0313175 (PMC11687784; doi:10.1371/journal.pone.0313175)
Supplement: Attachment — Submitted filename: Revision of PONE.docx [file pone.0313175.s003.docx]

**Revision of PONE-D-24-06321**

I would like to congratulate the authors of the manuscript for their work, as I believe that both its subject matter and execution represent a significant contribution to the nursing scientific community, both in clinical and research areas. Therefore, we now make a series of minor observations to ensure that the study meets the quality standards of PLOS ONE.

In general terms, I recommend that the authors review the writing style of the manuscript to ensure compliance with the spelling recommendations in scientific publications.

**Introduction**

(Lines 109-113) I believe it is necessary to include some information regarding the typical structure of OSCEs, such as the number of scenarios, recommended duration, the context in which they are usually conducted, evaluation forms, etc. The authors recommend that they consult Harden's original reference for this purpose.

(Lines 114-116) I think it is relevant to describe what is already known about reliability and agreement in the checklists used in the intensive care unit, to contextualize the need to conduct one concerning an OSCE in this setting.

**Methodology**

I would recommend that the authors include a figure based on a flow chart detailing each stage of the study.

(Lines 145-147) The authors mention that the recruitment was carried out at a conference, suggesting convenience sampling. However, this is not explicitly mentioned, which I believe would add transparency to the article.

(Lines 153-159) In addition to mentioning the COSMIN checklist, I think it is important to mention the statistical parameters used and the value at which they were set to calculate the minimum sample size required for the study.

(Lines 161-168) I would further detail the most relevant characteristics of the simulation scenarios (duration, simulated clinical situation, necessary material and human resources, characteristics of the simulators, etc.).

(Lines 170-172) It is necessary to specify the methodology followed for developing the initial version of NEUMOBACT (it is not mentioned whether it was a literature review, focus groups with thematic analysis and triangulation, etc.).

(Lines 202-209) It would be important for the authors to provide the DOI or registration ID of the study in registries such as the Open Science Framework.

**Results**

(Lines 235-237) I believe it is relevant to detail the methodology followed for developing the modified version of NEUMOBACT (it is not mentioned whether it was a literature review, focus groups with thematic analysis and triangulation, etc.).

**Discussion**

(Line 278) I would mention future perspectives for upcoming research related to the topic. I would also highlight the potential benefit of applying simulated learning methodologies at the undergraduate and postgraduate levels, such as MAES or OSCEs with validated checklists, to improve patient safety and quality of care.

**Limitations**

(Lines 280-288) I believe it is important to incorporate more citations to support the claims made by the authors. I also think they should mention the geographical limitation of NEUMOBACT validation, as well as possible recommendations for its use in contexts outside of Spanish-speaking ones.

**References**

From reference number 10 onward, there is an indentation that is not present in references 1-9, so I recommend that the authors review the format used.
