# Supplementary material for: Interrater reliability and agreement of the NEUMOBACT checklist about infection-prevention performance of intensive care nurses in simulation-based scenarios
Source: PLoS One. 2024 Dec 31;19(12):e0313175. doi: 10.1371/journal.pone.0313175 (PMC11687784; doi:10.1371/journal.pone.0313175)
Supplement: Attachment — Submitted filename: R2_Response to Reviewers.docx [file pone.0313175.s004.docx]

Authors: Thank you for the opportunity to review the manuscript. We have now addressed the editor's and reviewers' comments. We have added the completed GRRAS checklist. We apologise for not having done so previously.

Journal Requirements:

Please review your reference list to ensure that it is complete and correct. If you have cited papers that have been retracted, please include the rationale for doing so in the manuscript text, or remove these references and replace them with relevant current references. Any changes to the reference list should be mentioned in the rebuttal letter that accompanies your revised manuscript. If you need to cite a retracted article, indicate the article’s retracted status in the References list and also include a citation and full reference for the retraction notice.

Authors: They have been rechecked for any errors.

Additional Editor Comments:

**Dear Authors,

This round of revisions have complied with the Reviewers comments, and this manuscript could be accepted for publication.

However, before this can be accepted formally, one Reviewer recommends to complete a formal change in the Methodology.

--> (Line 144) The authors have added the GRRAS checklist as supplementary material but not completed it.

Please, complete the GRASS checklist with appropriate information, and also your manuscript if necessary, and address it again.

Best regards.**

Authors: We apologise for the error. We have attached the completed GRRAS checklist.

[Note: HTML markup is below. Please do not edit.]

Reviewers' comments:

Reviewer's Responses to Questions

**Comments to the Author**

1. If the authors have adequately addressed your comments raised in a previous round of review and you feel that this manuscript is now acceptable for publication, you may indicate that here to bypass the “Comments to the Author” section, enter your conflict of interest statement in the “Confidential to Editor” section, and submit your "Accept" recommendation.

Reviewer #1: All comments have been addressed

Reviewer #2: (No Response)

Authors: Thank you for your efforts in reviewing our manuscript.

2. Is the manuscript technically sound, and do the data support the conclusions?

The manuscript must describe a technically sound piece of scientific research with data that supports the conclusions. Experiments must have been conducted rigorously, with appropriate controls, replication, and sample sizes. The conclusions must be drawn appropriately based on the data presented.

Reviewer #1: Yes

Reviewer #2: Yes

3. Has the statistical analysis been performed appropriately and rigorously?

Reviewer #1: Yes

Reviewer #2: Yes

4. Have the authors made all data underlying the findings in their manuscript fully available?

The [PLOS Data policy](http://c05y1x9s.r.us-east-2.awstrack.me/L0/http:%2F%2Fwww.plosone.org%2Fstatic%2Fpolicies.action%23sharing/1/010f019276bfc1e9-118f4a76-c8de-4063-a9d4-f1d6615ac3b2-000000/OicaaeNEE5fyAVBrDYeCchUFx2M=180) requires authors to make all data underlying the findings described in their manuscript fully available without restriction, with rare exception (please refer to the Data Availability Statement in the manuscript PDF file). The data should be provided as part of the manuscript or its supporting information, or deposited to a public repository. For example, in addition to summary statistics, the data points behind means, medians and variance measures should be available. If there are restrictions on publicly sharing data—e.g. participant privacy or use of data from a third party—those must be specified.

Reviewer #1: Yes

Reviewer #2: Yes

5. Is the manuscript presented in an intelligible fashion and written in standard English?

PLOS ONE does not copyedit accepted manuscripts, so the language in submitted articles must be clear, correct, and unambiguous. Any typographical or grammatical errors should be corrected at revision, so please note any specific errors here.

Reviewer #1: Yes

Reviewer #2: Yes

6. Review Comments to the Author

Please use the space provided to explain your answers to the questions above. You may also include additional comments for the author, including concerns about dual publication, research ethics, or publication ethics. (Please upload your review as an attachment if it exceeds 20,000 characters)

Reviewer #1: I extend my congratulations to the authors for the excellent work they have done, as well as for their efforts in incorporating the suggested recommendations. These adjustments, in my opinion, have significantly improved the overall quality of the article, ensuring that it now aligns more closely with the standards and expectations of the journal. In this regard, I would like to emphasize one final suggestion: I strongly encourage the authors to consider the benefits of registering the studies conducted in dedicated registries designed for this purpose.

Authors: Thank you for your efforts in reviewing our manuscript. We agree with their recommendations and will take them into account for further studies, which will be registered before being sent for peer review

Reviewer #2: I would like to thank the authors for their prompt response, as well as for the work of reviewing and implementing most of the suggestions in one way or another. However, I would still like to comment a little note:

Methodology

(Line 144) The authors have added the GRRAS checklist as supplementary material but not completed.

Authors: We apologise for the error. We have attached the completed GRRAS checklist.

Without further ado, I hope that the article can be accepted soon.

Authors: Thank you for your efforts in reviewing our manuscript.

7. PLOS authors have the option to publish the peer review history of their article ([what does this mean?](https://c05y1x9s.r.us-east-2.awstrack.me/L0/https:%2F%2Fjournals.plos.org%2Fplosone%2Fs%2Feditorial-and-peer-review-process%23loc-peer-review-history/1/010f019276bfc1e9-118f4a76-c8de-4063-a9d4-f1d6615ac3b2-000000/-jsTvgbJJ0kVtyJ2orWwaXdpFHY=180)). If published, this will include your full peer review and any attached files.


If you choose “no”, your identity will remain anonymous but your review may still be made public.


**Do you want your identity to be public for this peer review?** For information about this choice, including consent withdrawal, please see our [Privacy Policy](https://c05y1x9s.r.us-east-2.awstrack.me/L0/https:%2F%2Fwww.plos.org%2Fprivacy-policy/1/010f019276bfc1e9-118f4a76-c8de-4063-a9d4-f1d6615ac3b2-000000/1P6MOrt04uFD9CMQuyXnIES-LFA=180).

Reviewer #1: No

Reviewer #2: No
